# Acaricides Resistance in Ticks: Selection, Diagnosis, Mechanisms, and Mitigation

**DOI:** 10.3389/fcimb.2022.941831

**Published:** 2022-07-06

**Authors:** Muhammad Kashif Obaid, Nabila Islam, Abdulaziz Alouffi, Alam Zeb Khan, Itabajara da Silva Vaz, Tetsuya Tanaka, Abid Ali

**Affiliations:** ^1^ Department of Zoology, Abdul Wali Khan University Mardan, Mardan, Pakistan; ^2^ Department of Chemistry, Abdul Wali Khan University Mardan, Mardan, Pakistan; ^3^ King Abdulaziz City for Science and Technology, Riyadh, Saudi Arabia; ^4^ Department of Pediatrics, Yale School of Medicine Yale University, New Haven, CT, United States; ^5^ Centro de Biotecnologia and Faculdade de Veterinária, Universidade Federal do Rio Grande do Sul, Porto Alegre, Brazil; ^6^ Laboratory of Infectious Diseases, Joint Faculty of Veterinary Medicine, Kagoshima University, Kagoshima, Japan

**Keywords:** acaricides, mitigation, resistance, SNPs, ticks

## Abstract

Ticks are blood-feeding ecto-parasites that have a cosmopolitan distribution in tropical and subtropical regions of the world. Ticks cause economic losses in the form of reduced blood, meat and dairy products, as well as pathogen transmission. Different acaricides such as organochlorines, organophosphates, formamidines (e.g. amitraz), synthetic pyrethroids, macrocyclic lactones, fipronil, and fluazuron are currently used sequentially or simultaneously to control tick infestations. Most acaricide treatments now face increasingly high chances of failure, due to the resistance selection in different tick populations against these drugs. Acaricide resistance in ticks can be developed in different ways, including amino acid substitutions that result in morphological changes in the acaricide target, metabolic detoxification, and reduced acaricide entry through the outer layer of the tick body. The current literature brings a plethora of information regarding the use of different acaricides for tick control, resistance selection, analysis of mutations in target sites, and resistance mitigation. Alternatives such as synergistic use of different acaricides, plant-derived phytochemicals, fungi as biological control agents, and anti-tick vaccines have been recommended to avoid and mitigate acaricide resistance. The purpose of this review was to summarize and discuss different acaricides applied for tick control, their mechanisms of action and resistance selection, genetic polymorphisms in their target molecules, as well as the approaches used for diagnosis and mitigation of acaricide resistance, specifically in *Rhipicephalus microplus* ticks.

## Introduction

Ticks are hematophagous ecto-parasites with global geographical distribution, especially in tropical and subtropical regions, where they parasitize terrestrial and semi-terrestrial vertebrates (Ali et al., 2019; Guglielmone et al., 2020). Ticks also serve as vector reservoirs for the spread of pathogens among their vertebrate hosts (Parola and Raoult, 2001). In the livestock industry, this results in significant losses due to host blood depletion, general discomfort and irritation, reduced dairy and meat production, suppression of immune functions, and damage to hides, among other negative impacts (Desta, 2016; Tabor et al., 2017).

Before the introduction of synthetic acaricides, different chemicals such as cotton-seed oil, fish oil, beaumont crude oil, the combination of lard oil with sulfur as well as lard oil with kerosene oil have been used on the host’s body surface to control tick infestations. In cattle, for example, the combination of kerosene oil with cotton-seed oil and sulfur, kerosene oil with cotton-seed oil, and crude petroleum alone have shown effectiveness against ticks (Mohler, 1906). Arsenic, introduced in 1895 (National Research Council, 1977; Angus, 1996), was the first acaricide widely used to control ticks and tick-borne diseases until the introduction of organochlorines (OCs), because it was cheap, stable, and water-soluble (Graham and Hourrigan, 1977; George et al., 2004). Arsenic was used to control tick infestations in different countries like Australia (Angus, 1996), South Africa (Matthewson and Baker, 1975), Mexico, and the USA (Drummond et al., 1964). In 1937, treatment failure due to the selection of arsenic resistant *Rhipicephalus microplus* was reported for the first time in Australia (Newton, 1967), and arsenic resistant *Rhipicephalus decoloratus* (Whitehead, 1958) as well as *Rhipicephalus appendiculatus* and *Rhipicephalus evertsi* (Matthewson and Baker, 1975) were documented in South Africa in 1940 and 1975, respectively.

The OCs, organophosphates (OPs), amitraz, synthetic pyrethroids (SPs), macrocyclic lactones (MLs), and fipronil are commonly used acaricides that act on the tick central nervous system through various mechanisms, affecting gamma-aminobutyric acid (GABA)-gated chloride channels, inhibitors of acetylcholine-esterase (AChEs), octopamine tyramine receptors, voltage-gated sodium channels, glutamate gated chloride channels (Glu-Cl), and GABA-receptors, respectively (Chen et al., 2007). Different acaricides have specific targets and different modes of action, which affects the reproduction, growth and survival of various tick species (Klafke et al., 2017; Klafke et al., 2019). Spraying, washing, pouring, and injections are the different methods for the application of acaricides on host animals (Food and Agriculture Organization, 2004; Higa et al., 2019). Incorrect dilution, inappropriate application, persistent use and overdosing are the main factors that hasten acaricide resistance selection in ticks (Aguilar-Tipacamu et al., 2011; Abbas et al., 2014). Certain species may be even more prone to develop acaricide resistance, due to favorable conditions created by their broad distribution and/or particularities of their life cycle (Guerrero et al., 2012a).

*Rhipicephalus annulatus*, *R. microplus*, and *Rhipicephalus australis* undergo rapid selection in response to selective pressure and higher concentration of acaricides (Burger et al., 2014). Different populations of *R. microplus* have been found resistant to all acaricides that have been used for their control, including: arsenics (Newton, 1967), OCs e.g. dichloro-diphenyl-trichloroethane (DDT) (Stone and Webber, 1960; Bandara and Karunaratne, 2017), OPs (Li et al., 2003; Miller et al., 2005), MLs e.g. ivermectin (Klafke et al., 2006; Perez-Cogollo et al., 2010a), formamidines e.g. amitraz (Miller et al., 2002), SPs (Guerrero et al., 2001; Kumar et al., 2013; Ghosh et al., 2015; Klafke et al., 2019), fipronil (Castro-Janer et al., 2010), and fluazuron (Reck et al., 2014). The surveillance of acaricide resistance in various regions is essential in order to adopt adequate treatment approaches. Globally the acaricide-resistant ticks identified through different diagnostic tests have been reported (**Table 1**).

**Table 1:** Historical and regional aspects of different acaricides, resistant ticks and diagnostic approaches.

Acaricides	Approximate Introduction Year	Region of the First Study	Tick Species	Diagnostic Test	Reference
Organochlorines	1946	Australia	*R. microplus*	AIT	Norris and Stone, 1956
		Australia	*R. microplus*	LIT, AIT	Stone and Meyers, 1957
		Australia	*R. microplus*	LIT, AIT	Stone and Webber, 1960
		India	*R. microplus*	LIT, AIT	Chaudhuri and Naithani, 1964
		South Africa	*R. appendiculatus*	LIT, AIT	Baker and Shaw, 1965
		Uganda	*R. decoloratus*; *R. evertsi*; *R. appendiculatus*	Larvae tests	Kitaka et al., 1970
		Tanzania	*R. microplus*; *R. decoloratus*; *Amblyomma (Am)* spp.	LPT	Kagaruki, 1991
		Ethiopia	*R. decoloratus*; *Rhipicephalus* spp.	LPT	Regassa and de Castro, 1993
		Mexico	*R. microplus*	LPT	Ortiz Estrada et al., 1995
		Australia	*R. microplus*	LPT	Hope et al., 2010
Organophosphates	1955	South Africa	*R. microplus*	LIT, AIT	Baker et al., 1979
		Zambia	*R. decoloratus*; *Rhipicephalus* spp. *Am. variegatum*	LPT	Luguru et al., 1987
		Venezuela	*R. microplus*	Spraying on hosts	Coronado, 1995
		Mexico	*R. microplus*	LPT	Ortiz Estrada et al., 1995
		Mexico	*R. microplus*	LPT	Rosario-Cruz et al., 1997
		Colombia	*R. microplus*	LIT	Benavides et al., 2000
		Mexico	*R. microplus*	LPT	Li et al., 2003
		USA	*R. microplus*	LPT	Miller et al., 2005
		Brazil	*R. microplus*	AIT	Baffi et al., 2007
		Argentina	*R. microplus*	LTT	Lovis et al., 2013
		Australia	*R. microplus*	LTT	Lovis et al., 2013
		USA	*R. microplus*	LPT	Busch et al., 2014
Formamidines (Amitraz)	1975	Brazil	*R. microplus*	LPT	Martins et al., 1995a
		Australia	*R. microplus*	Molecular characterization	Baxter and Barker, 1999
		Mexico	*R. microplus*	LIT	Céspedes et al., 2002
		USA/Brazil	*R. microplus*	LPT	Li et al., 2004
		New Caledonia	*R. microplus*	LPT	Ducornez et al., 2005
		USA	*R. microplus*	Molecular characterization	Chen et al., 2007
		Argentina	*R. microplus*	LTT	Lovis et al., 2013
		USA	*R. microplus*	LPT	Busch et al., 2014
		Uruguay	*R. microplus*	LPT, LIT	Cuore and Solari, 2014
		India	*R. microplus*	AIT	Singh et al., 2015a
		South Africa	*R. microplus*	LPT	Baron et al., 2015
		Uganda	*R. decoloratus*; *Rhipicephalus* spp.	LPT	Vudriko et al., 2016
		Zimbabwe	*R. microplus*	Molecular characterization	Sungirai et al., 2018
Synthetic Pyrethroids	1977	Australia	*R. microplus*	LIT	Nolan et al., 1979
		New Caledonia	*R. microplus*	LPT	Brun, 1992
		New Caledonia	*R. microplus*	LPT	Beugnet and Chardonnet, 1995
		Brazil	*R. microplus*	LPT	Martins et al., 1995a
		Mexico	*R. microplus*	LPT	Ortiz Estrada et al., 1995
		Mexico	*R. microplus*	LPT	Rosario-Cruz et al., 1997
		USA	*R. microplus*	LPT	Davey and George, 1998
		Colombia	*R. microplus*	LIT	Benavides et al., 2000
		Panama	*R*. *sanguineus*	LPT	Miller et al., 2001
		South Africa	*R. decoloratus*	LIT	Mekonnen et al., 2002
		Argentina	*R. microplus*	LPT	Mangold et al., 2005
		Iran	*Hyalomma (Hy)* spp.	LPT	Khaladj et al., 2007
		Pakistan	*Hy. anatolicum*	AIT	Sajid et al., 2009
		India	*R. microplus*	LPT, AIT	Sharma et al., 2012
		Australia	*R. microplus*	LTT	Lovis et al., 2013
		South Africa	*R. microplus*	LTT	Lovis et al., 2013
		France	*R. geigyi*	LPT	Adakal et al., 2013
		USA	*R. microplus*	LPT	Busch et al., 2014
		Uganda	*R. decoloratus*; *Rhipicephalus* spp.	LPT	Vudriko et al., 2016
Macrocyclic lactones	1981	Brazil	*R. microplus*	AIT	Martins and Furlong, 2001
		Brazil	*R. microplus*	LIT	Klafke et al., 2006
		Pakistan	*Hy. anatolicum*	AIT	Sajid et al., 2009
		Mexico	*R. microplus*	LIT	Perez-Cogollo et al., 2010a
		Uruguay	*R. microplus*	LIT	Castro-Janer et al., 2011
		Switzerland	*R. microplus*	LTT	Lovis et al., 2011
		USA	*R. microplus*	LPT	Busch et al., 2014
		India	*R. microplus*	LIT	Singh et al., 2015b
		Colombia	*R. microplus*	LIT	Villar et al., 2016
		Egypt	*R. annulatus*	LIT	El-Ashram et al., 2019
		Brazil	*R. sanguineus*	LPT, LIT	Becker et al., 2019
Fipronil	1987	Uruguay	*R. microplus*	—	Cuore et al., 2007
		Uruguay	*R. microplus*	LIT	Castro-Janer et al., 2009
		USA/Mexico	*R. microplus*	LPT	Miller et al., 2013
		USA	*R. microplus*	LPT	Busch et al., 2014
		Brazil	*R. microplus*	AIT	Klafke et al., 2017
		India	*R. annulatus* *Haemaphysalis bispinosa*	AIT	Ravindran et al., 2018
		Pakistan	*Hy. anatolicum*	LIT, AIT	Kamran et al., 2021
		Haryana (India)	*Hy. anatolicum*	LPT	Gupta et al., 2021
		Turkey	*R. sanguineus*	LPT	Samed et al., 2021
Fluazuron	1994	Brazil	*R. microplus*	LPT, AIT, field trials and AFA	Reck et al., 2014

AIT, Adult immersion test; LIT, Larval immersion test; LPT, Larval packet test; LTT, Larval tarsal test; AFA, Artificial feeding assays; R., Rhipicephalus; Hy., Hyalomma; Am., Amblyomma; Ha., Haemaphysalis.

In recent years, a number of reviews have discussed different aspects of acaricide resistance, including identification of resistant populations, development of novel diagnostic methods, effects of resistance in tick metabolism, environment pollution, evolution and spread of resistant populations, and actions to mitigate risks related to acaricide resistance (George, 2000; George et al., 2004; Abbas et al., 2014; Benelli et al., 2017; Banumathi et al, 2017; Nwanade et al., 2020; Agwunobi et al., 2021). Genetic mutations in ticks frequently result in the selection of acaricide resistance (de Oliveira Souza Higa et al., 2015), causing conformational changes in the target site, enhanced acaricide metabolism, or reduced ability by the drug to penetrate the tick’s outer defensive layers (Guerrero et al., 2012a). A comprehensive understanding on acaricides regarding their mode of action, resistance development, and single nucleotide polymorphisms (SNPs) in the target sites is needed to mitigate acaricides resistance in different tick species. This review paper aims to summarize and critically discuss different acaricides applied for tick control, with focus on mode of action, genetic polymorphisms on target sites, diagnostic approaches, and mitigation strategies regarding acaricides resistance.

## Definition and Types of Acaricide Resistance

Tick resistance against acaricides is described as “the specific heritable trait(s) in a population of ticks selected as a result of the population’s interaction with an acaricide”. The number of ticks that remain alive after exposure to the specific applied concentration of acaricides increases significantly (Rodriguez-Vivas et al., 2018). The mutated genes, inherited from the survivor ticks, are initially uncommon and rarely occur in the tick population, but increase in frequency with time. Acquired resistance is thus defined as “a resistance that results from heritable declines in drug susceptibility with the passage of time” (Chapman, 1997), which in turn results in phenotypic resistance (Esteve-Gasent et al., 2020). The term tolerance refers to the ability of a parasite to survive exposure to a specific drug dose that would usually be considered effective. Resistance across different active chemical ingredients having similar mechanisms of actions is known as cross-resistance (Li et al., 2003, Li et al., 2005a).

**Figure 1:**
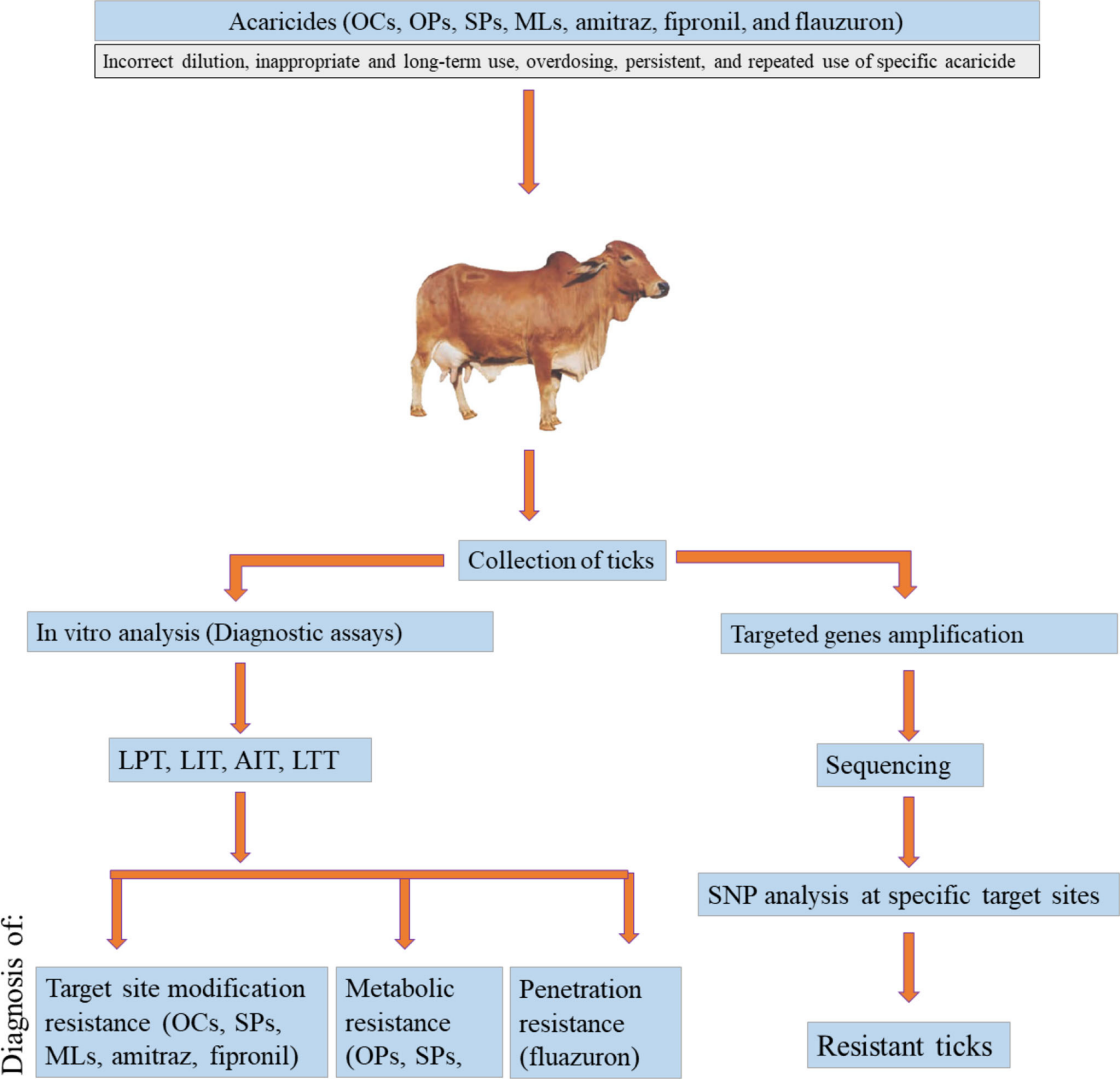
Flow chart of tick resistance diagnostics.

Three main forms of acaricide resistance are generally known (**Figure 1**). Metabolic resistance is achieved through acaricide detoxification by the metabolic activity of enzymes such as cytochrome P-450s (CYP), esterases, and glutathione S-transferase (GST) (Hemingway and Ranson, 2000; Guerrero et al., 2012a). Resistance developed through conformational changes in the acaricide target site in neuronal enzymes and receptors, leading to impaired interaction between the drug and the target, is termed “target site modification resistance” (Coles and Dryden, 2014). The decreased access of acaricides to the internal body environment due to modifications in the tick outer layer (exoskeleton) is called reduced penetration resistance (Schnitzerling et al., 1983; Guerrero et al., 2012a).

## Historical Perspective

**Figure 2:**
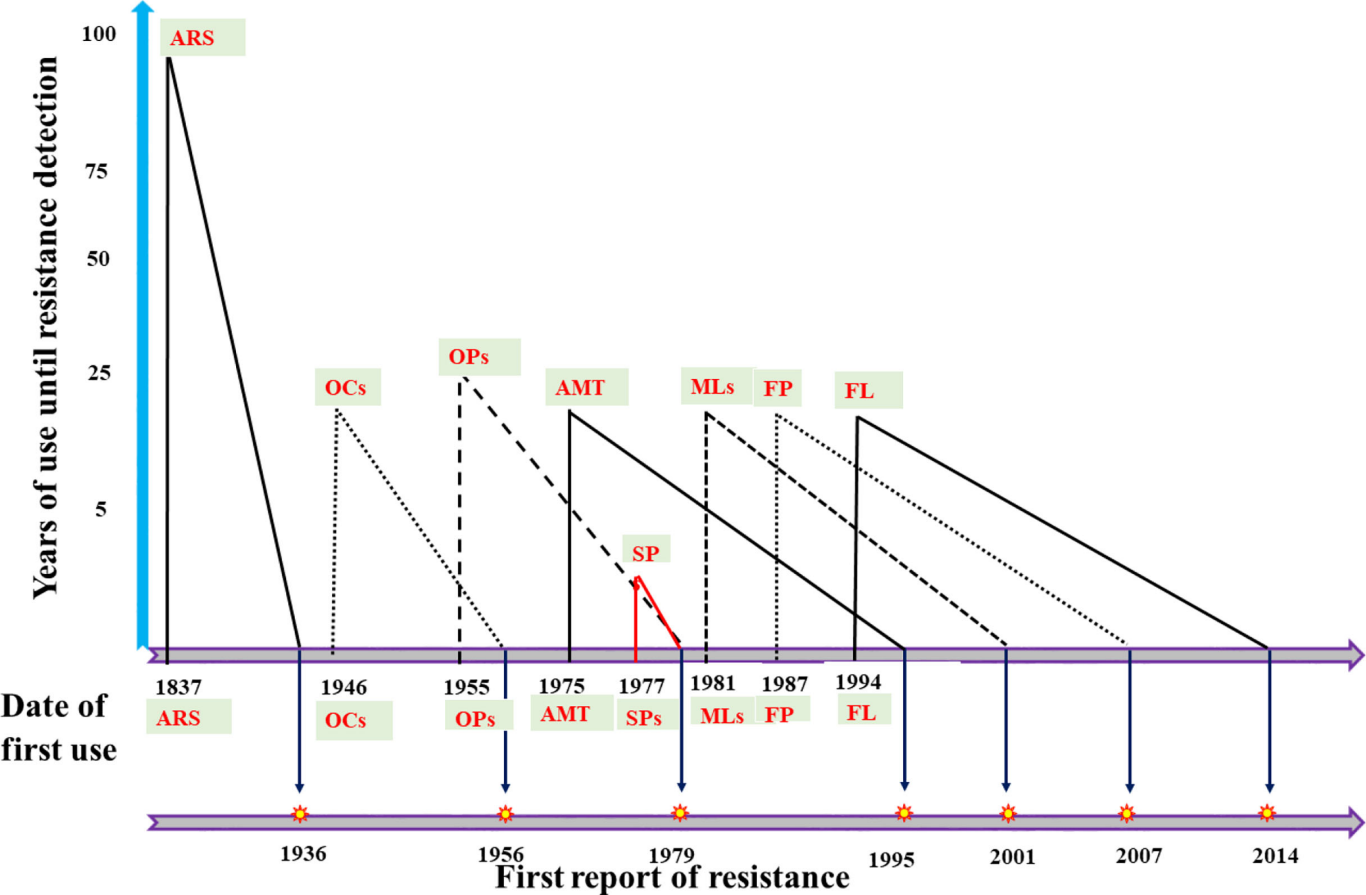
Historical representation of acaricide introduction and first reports of resistant ticks.

**Figure 2** presents a general timeline showing different acaricides regarding their year of introduction and the first reports of resistance. Organochlorines (OCs) were the first commercially viable synthetic acaricides, and were introduced in 1946. Dichloro-diphenyl-trichloroethane (DDT) and benzene hexachloride (BHC) were the first OCs used as acaricides (Maunder, 1949; Whitnall et al., 1951), and resistance against both drugs was documented in *R. microplus* as early as 1960 in Australia (Stone and Webber, 1960). The organophosphates (OPs) were introduced approximately in 1955 (dioxathion), and resistance against this acaricide was recorded for the first time in 1987 in *R. decoloratus* and *Amblyomma variegatum* from Zambia (Luguru et al., 1987). Formamidines were introduced in the early 1960s (Hollingworth, 1976), while amitraz was registered for the first time in 1975, just 2 decades before the first amitraz-resistant *R. microplus* ticks were reported in Brazil (Martins et al., 1995a). Amitraz-resistant *R. microplus* ticks were confirmed in 2001 in Mexico (Céspedes et al., 2002). Synthetic pyrethroids (SPs) were introduced in 1977, while resistance against SPs was observed for the first time just 2 years later, in *R. microplus* from Australia (Nolan et al., 1979). Macrocyclic lactones (MLs), in turn, were introduced in 1981, and resistance against MLs was recorded for the first time in 2001 in *R. microplus* from Brazil (Martins and Furlong, 2001). Fipronil was introduced in 1987, and resistant *R. microplus* ticks were reported for the first time in 2007 in Uruguay (Cuore et al., 2007). The growth regulator fluazuron was introduced in 1994 (Junquera et al., 2019), and similar to other acaricides, first reports of resistant ticks arised 20 years later, in 2014, in *R. microplus* from Brazil which were already resistant to all six types of available acaricides (Reck et al., 2014).

## Diagnostic Tests

Monitoring acaricide resistance in the field tick population is necessary for optimal and strategic use, slowing resistance selection, and surveillance of different acaricide resistant ticks. Diagnosis of resistance against different acaricides is essential and urgently required through efficient and inexpensive tests which should be easily followed (Sabatini et al., 2001).

The larval packet test (LPT), developed in 1962, uses acaricide-treated filter paper packets to incubate the tick larvae (Stone and Haydock, 1962). This test has been used to survey and diagnose resistance against OPs and SPs (Guerrero et al., 2014). The LPT takes 5–6 weeks to be completed, thus it is restricted by the lead time until results are available. The larval immersion test (LIT) was developed in 1966 (Shaw, 1966). In this assay, larvae are immersed in the test acaricide solution prepared at the applicable concentrations, to check the mortality rate and to obtain resistant ticks. This test is usually employed to characterize resistance against amitraz and MLs (Rodriguez-Vivas et al., 2006b; Perez-Cogollo et al., 2010a). In adult immersion test (AIT), which has been developed in 1973 (Drummond et al., 1973) and is currently applied in different studies, engorged female ticks are immersed in acaricide solutions prepared according to the tested concentrations (Whitnall et al., 1951). Despite broadly used for different acaricides, the AIT is not suitable to test for resistance against amitraz, which is one of the most commonly used acaricides in Mexico (Jonsson and Hope, 2007). The larval tarsal test (LTT) was established for the first time in Switzerland in 2011. It is a highly sensitive and time-efficient *in vitro* test, and has been used to determine resistance levels in *R. microplus*, as well as other ixodid ticks (Lovis et al., 2011).

## Organochlorines

Organochlorines include ethane-derived chlorates such as DDT, cyclodienes (chlordane, aldrin, dieldrin, endrin, heptachlor, and toxaphene), and compounds related to hexa-chlorocyclohexane such as lindane (Jayaraj et al., 2016). Organochlorines were demonstrated to cause mortality, suppression of fertility as well as inhibition of egg hatching in *R. annulatus* and *Haemaphysalis bispinosa* engorged female ticks (Ravindran et al., 2018). Resistance against toxaphene in *R. decoloratus*, *R. appendiculatus*, and *R. evertsi* has been documented (Kitaka et al., 1970). Organochlorines have a wide range of activity against arthropods (ticks and insects), but have harmful impacts as may be found in the environment, milk, meat as well as in the fat of animal hosts after their application (Beugnet and Franc, 2012). This lipophilic property was the main drawback of OCs, for which its use against ticks was banned (Graf et al., 2004).

## Organochlorines Mechanism of Action and Resistance

The target site for DDT is the voltage-gated sodium channels of the axon, and resistance is caused by mutations in the amino acid at the binding site (Ware and Whitacre, 2004). The targets for dieldrin are GABA-gated chloride channel receptors (Hope et al., 2010). The drug modifies the channel properties, causing hyperexcitation by inhibiting the entry of chloride ions into the nerve, ultimately leading to the death of ticks (Ozoe et al., 2010). Cyclodienes cause mutations (single nucleotide polymorphisms, or SNPs within the GABA-gated chloride channel gene that result in the selection of tick resistance (Ffrench-Constant et al., 2000). Nucleotide mutations (non-synonymous and synonymous) in *R. microplus* genes targeted by or associated with specific acaricides have been identified. Mutations related to OCs use in *R. microplus* were different from the observed for certain insects, however they were located in the same second transmembrane protein domain of the GABA-gated channel (TM-2). The effect of this mutation was unknown, but the modified TM-2 may alter the channel properties, inhibiting the entry of chloride ions into the nerve (Corley et al., 2012), and resulting in the insensitivity of the dieldrin’s target site (Ffrench-Constant et al., 2000). Polymorphisms had also been identified in the voltage-gated sodium channels of nerve axons, a target of DDT, which causes the channel’s closure after depolarization and results in a consecutive leakage of sodium ions across the membrane of the neuron (Holan, 1969) (**Table 2**).

**Table 2:** Nucleotide and amino acid substitutions in acaricide target genes in *Rhipicephalus microplus* ticks.

Acaricides	Target	Domain/Location	Nucleotide Mutations	Amino Acid Substitutions	NS/S	References
Organochlorides	GABA-gated chloride channel gene	TM-2	A-868-C	T-290-L	NS	Hope et al., 2010
			C-869-T			
	Voltage-gated sodium channel gene (only for dichloro-diphenyl-trichloroethane)	Domain-II	G-215-T	G-72-V	NS	Bandara and Karunaratne, 2017
Organophosphates	AChE genes	AChEs 1	**-**	**-**	**-**	**-**
		AChEs 2	G-138-A	–	S	Ghosh et al., 2015
			G-889-A	V-297-I	NS	
			T-1090-A	S-364-T	NS	
			C-1234-T	H-412-Y	NS	
			G-1403-A	R-468-K	NS	
		AChEs 3	A-366-C	I-48-L	NS	Temeyer et al., 2007
			A-384-G	I-54-V	NS	
			G-481-A	R-86-Q	NS	
			G-633-A	V-137-I	NS	
			C-1700-G	I-492-M	NS	
			A-1866-G	T-548-A	NS	
			A-366-C	I-48-L	NS	Singh et al., 2016
			A-384-G	I-54-V	NS	
			G-481-A	R-86-Q	NS	
			T-212-C	V-71-A	NS	
			A-231-G	I-77-M	NS	
			T-235-C	S-79-P	NS	
Formamidines (Amitraz)	Octopamine tyramine gene	TM-1	A-181-T	I-61-F	NS	Corley et al., 2013; Takata et al., 2020
			A-22-C	T-8-P	NS	Chen et al., 2007
			T-65-C	L-22-S	NS	
			A-157-C	T-8-P	NS	Baron et al., 2015
			A-178-G	I-15-V	NS	
			A-193-G	T-20-A	NS	
			T-200-C	L-22-S	NS	
			T-123-C	–	S	Jonsson et al., 2018
			C-126-T	–	S	
			A-181-T	I-61-F	NS	
			T-185-C	I-62-T	NS	
			A-225-G	–	S	
			A-263-C	Y-88-S	NS	
			C-264-A	Y-88-S	NS	
Synthetic Pyrethroids	Voltage-gated sodium channel gene	Domain-I	–	–	–	
		Domain-II	C-190-A	L-64-I	NS	Morgan et al., 2009; Domingues et al., 2012; Lovis et al., 2012; Ghosh et al., 2015; Robbertse et al., 2016; Nagar et al., 2018; Janer et al., 2021
			C-148-T	L-50-F	NS	Stone et al., 2014
			G-184-C	G-62-R	NS	
			C-189-A	–	S	
			C-190-G	L-64-V	NS	
			T-170-C	M-57-T	NS	Klafke et al., 2019
			G-184-C	G-62-R	NS	
			C-190-A	L-64-I	NS	
			G-215-T	G-72-V	NS	
			G-214-T	G-72-V	NS	Jonsson et al., 2010a; Lovis et al., 2012
		Domain-III	C-2130-T	–	S	Stone et al., 2014
			T-2134-A	F-712-I	NS	He et al., 1999; Stone et al., 2014
			T-2134-A		NS	Klafke et al., 2019; Janer et al., 2021
			C-2136-A	F-712-L	NS	
		Domain-IV	–	–	–	–
Macrocyclic lactones	Glu-Cl channel gene		T-546-C	NR	-	Aguilar-Tipacamú et al., 2016
			T-575-C	NR	-	
Fipronil	GABA-gated chloride channel gene	TM-2	A-868-C	T-290-L	NS	Janer et al., 2019; Janer et al., 2021
			C-869-T			
			T-843-A	S-281-T	NS	
			G-858-C	A-286-S	NS	
			G-858-C	A-286-L	NS	
		TM3	G-949-A	V-317-I	NS	
			A-982-G	T-328-A	NS	
			G-985-T	A-329-S	NS	

GABA, Gamma-aminobutyric acid; NS, non- synonymous; AChEs, Acetylcholine esterases; S, synonymous; Glu-Cl, Glutamate gated chloride; NR, not reported in paper.

## Organophosphates

Organophosphates acaricides were introduced in the market mainly for the control of OC-resistant *Rhipicephalus* ticks, which were alarmingly increasing in numbers (Shaw, 1970). This class of acaricides include coumaphos, chlorpyrifos, chlorfenvinphos, diazinon, dioxathion, and ethion (Abbas et al., 2014; Adeyinka et al., 2018). From 1950s to the early 1970s, the OPs were used for tick control in Australia. They were used in large amounts in the national tick eradication program in Mexico between 1974 and 1984 (Trapaga, 1989).

*Rhipicephalus microplus* resistance against different OPs (coumaphos, chlorpyrifos, and chlorfenvinphos) have been recorded (Rosario-Cruz et al., 2009), with the first observations coming from South Africa in 1979 (Baker et al., 1979), then Mexico in 1980 (Ortiz Estrada et al., 1995). Organophosphates resistance in *R. microplus* is attributed to increased esterase enzyme activity (Rosario-Cruz et al., 2005), and different microarray techniques have been used to detect resistance against OPs, including coumaphos, in *R. microplu*s (Saldivar et al., 2008). Resistant homozygous *R. microplus* (strain Tuxpan) were experimentally obtained after the application of OP over seven successive generations (Foil et al., 2004).

Organophosphates do not accumulate in fatty tissues of animals like OCs, however, OPs are more toxic to humans than OCs (Chattopadhyay et al., 2004). The use of OPs poses health risks especially to agricultural and manufacturing workers, and children. Organophosphates target the central nervous system (CNS) and cause restlessness, headache, drowsiness, confusion, slurred speech, emotional lability, psychosis, ataxia (Kwong, 2002). Cardiovascular, respiratory, gastrointestinal, sensory, CNS, and motor systems are the most affected by OPs, with life-threatening outcomes (Collombet, 2011).

## Organophosphates Mechanism of Action and Resistance

**Figure 3:**
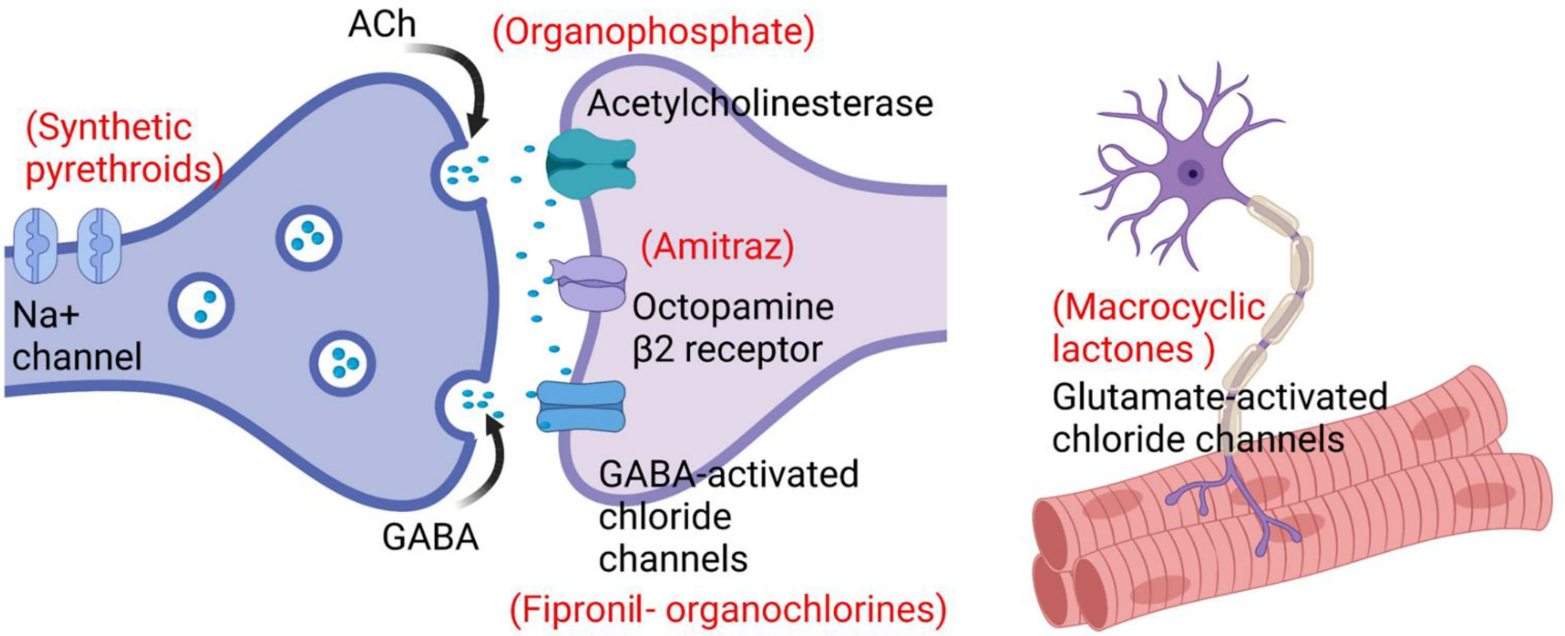
Target sites and mechanisms of action of acaricides.

A key enzyme (AChEs), necessary for nervous system functions in *R. microplus*, is the target site for OPs (**Figure 3**), as well as carbamates (carbaryl and promacyl) (Li et al., 2003; Temeyer et al., 2013). In the presence of a cholinesterase inhibitor, the enzyme would not be available to break down acetylcholine, resulting in a continuous neuron firing, which in turn causes overstimulation of the nervous system and lead to death (Temeyer et al., 2007).

Mechanisms of resistance to OPs include the modifications of the target site in the AChEs gene, carboxylesterase (CE) gene, and metabolic detoxification (Kumar, 2019). Organophosphates may interact with esterases found in the integument layers of *R. microplus*, resulting in the overexpression of esterases in larvae as well as in adult *R. microplus*, and consequent resistance to OPs (Villarino et al., 2001). Even though tick resistance to OPs is known to occur mostly through changes in metabolic activities and conformational changes in AChEs (Pruett, 2002; de Oliveira Souza Higa et al., 2015). Post-translational modifications in AChEs proteins have also been suggested as resistance mechanisms (Baxter and Barker, 1998).

The SNPs associated with OPs could be detected in three genes potentially coding for AChEs in *R. microplus* ticks: BmAChE-1 (Baxter and Barker, 1998), BmAChE-2 (Baxter and Barker, 2002), and BmAChE-3 (Temeyer et al., 2009). Searches for mutations in BmAChE-1 and BmAChE-2 associated with OP-resistance could not reveal a clear link to any of the identified amino acid substitutions. All six detected mutations were found in combination with each other except R-86-Q (Temeyer et al., 2007). These mutations are responsible for the insensitivity of *R. microplus* ticks to OPs (**Table 2**).

## Formamidines

Formamidines were introduced as alternatives to OPs after their failure. This class of acaricides includes chlordimeform, clenpyrin, chloromethiuron, amitraz, and cymiazole (Hollingworth, 1976). Chlordimeform was used in cattle’s vats to restore the effectiveness of OPs against OP-resistant *R. microplus* in Australia. Formamidine acaricides have been suggested to have a harmful impact on tick CNS, by targeting octopamine tyramine receptors (Dudai et al., 1987), and also inhibit the activity of monoamine oxidase enzymes in *R. microplus* (Schuntner and Thompson, 1977). The effectiveness of amitraz as an acaricide has been proven for several tick species including *R. microplus* and *R. annulatus* (Davey et al., 1984). The control efficacy of amitraz was more than 97%, and resulted in weight loss as well as reduced egg production in female ticks. Amitraz was used for rapid removal (within hours) of larvae, nymphs as well as adult ticks from the host (Davey et al., 1984).

In insects, octopamine is present in peripheral and central nervous systems, being involved in proprioception, vision and complex behaviors like flying, walking, and learning (Blenau and Baumann, 2001). In ticks, the putative octopamine receptor has been identified (Chen et al., 2007), and classified as OCT/Tyr receptor in phylogenetic studies (Corley et al., 2013; Baron et al., 2015). The examined gene was initially described as an octopamine-like G-protein-coupled receptor (GPCR) (Baxter and Barker, 1999; Baron et al., 2015; Robbertse et al., 2016). The GPCRs are known to have a wide range of important roles in eukaryotes, including physiology, reproduction, growth and development, stress responses, feeding, and behavior. They have seven-transmembrane proteins which relay signals in the extracellular environment to the intracellular second messenger system by binding to heterotrimeric G-proteins, adenylate cyclase, cAMPs, and protein kinases (Liu et al., 2021). Thus, these receptors transfer signals to second messenger pathways upon interaction with octopamine (Blenau and Baumann, 2001). Among all acaricides used for tick control in Uganda, 37% of the formulations were composed of amitraz (Vudriko et al., 2016), while this acaricide was the control method of choice in 27% of the surveyed farms in Zambia (Muyobela et al., 2015). Amitraz was effective in controlling ticks, however, resistance against amitraz was detected in *R. microplus* and other *Rhipicephalus* species in Australia, South America, and South Africa (Li et al., 2004, Li et al., 2005a; Jonsson and Hope, 2007; Vudriko et al., 2016). The selection of tick resistance against amitraz is a serious problem that attracted urgent attention. It has been observed that the chances of resistance selection become greater due to the excessive use of amitraz against *R. microplus* (Chen et al., 2007), and *Hyalomma anatolicum* (Jyoti et al., 2019). Research on the ovicidal activity of amitraz against *R. sanguineus* eggs has shown that one-day old eggs are more sensitive to amitraz than 10-12-day old eggs (Davey et al., 1989).

Drowsiness in animals, dry skin, and a harsh hair coat are frequent side effects of amitraz applications. It can cause toxicity in both animals and humans when swallowed, inhaled, or applied to the animal’s skin in high doses (Aydin et al., 1997; Phetudomsinsuk et al., 2014). It is carcinogenic in nature and was removed from the market in 1976 (Ware and Whitacre, 2004).

## Formamidines Mechanism of Action and Resistance

Octopamine receptors are found in acari, which makes it a suitable specific target site for acaricides (**Figure 3**), but no octopamine receptors have been described in mammalian cell membrane (Evans and Gee, 1980; Chen et al., 2007). The mechanism of action of amitraz is based on its toxicity-inducing interaction with octopamine receptors. Resistance in *R. microplus* might occur *via* conformational changes caused by mutations in the gene encoding octopamine receptor (Chen et al., 2007). Four putative amitraz resistance mechanisms have been described, including (1) insensitivity of octopamine tyramine receptor, (2) insensitive β-adrenergic octopamine receptors (BAOR), (3) enhanced expression of monoamine oxidase, and (4) the activity of ATP binding transporters (ABC-transporters) (Jonsson et al., 2018). The ABC-transporters act by pumping toxins out of the cell, and serving as a defense mechanism against acaricides in *R. microplus* (Pohl et al., 2011).

Amitraz resistance has been described as a complicated multigenic trait, with recessive resistance alleles (Li et al., 2004, Li et al., 2005a). Nucleotide mutations were identified in the octopamine tyramine receptor gene among resistant *R. microplus* ticks. All the polymorphisms in GPCR target sites were connected with amitraz resistance in *R. microplus*, and resulted in the insensitivity of the target (Baron et al., 2015) (**Table 2**).

## Synthetic Pyrethroids

Synthetic pyrethroids are chlorinated or brominated halogenated esters of a chrysanthemic acid isomer and synthetic alcohol molecule. Synthetic pyrethroids were introduced in the late 1970s, after the identification of resistance against formamidines in *R. microplus*. Permethrin was registered and authorized for use by the US Environmental Protection Agency (US Environmental Protection Agency [USEPA], 2006). Synthetic pyrethroids comprised about 17% of the total acaricides used at the beginning of the 21^st^ century (Davies et al., 2007). Different SPs can be categorized as type-I and type-II, according to the presence or absence of a cyano group in the alcohol portion (Soderlund and Bloomquist, 1989). Cypermethrin, flumethrin, cyhalothrin, and cyfluthrin are other common SPs used to control ticks (Bonnefoy et al., 2008). Permethrins were the first SPs used as acaricides, followed by cypermethrin and deltamethrin (Davies et al., 2007).

The CYP enzyme family controls the metabolism and detoxification of endogenous and exogenous cell damaging chemicals such as plant toxins, drugs, and pesticides in many arthropods (Kasai, 2004). A study regarding gene expression of CYP and CE related to detoxification of SPs in acaricide-resistant *R. microplus* showed higher levels of CYP expression in pyrethroid-resistant ticks as compared to susceptible ticks (Cossío-Bayúgar et al., 2018). Resistance against pyrethroid and propetamphos was observed in *R. bursa* in Iran, and found to be related to the metabolic activities of esterases, CYP, and GST enzymes (Enayati et al., 2010).

Commercially available SPs like deltamethrin and cypermethrin did not strongly induce resistance in *Hy. anatolicum* and *R. microplus* in India (Sharma et al., 2018). Reduction in egg masses of *R. microplus* upon the use of different concentrations of deltamethrin and cypermethrin was observed (Godara et al., 2019). A low to moderate level of resistance to λ-cyhalothrin and cypermethrin, respectively, in *R. annulatus* from Iran (Ziapour et al., 2017). Further, *R. sanguineus* eggs were more sensitive to cis-cypermethrin and cypermethrin than to coumaphos (Bicalho et al., 2001), although when nymphs and unfed females were treated with coumaphos, cis-cypermethrin, deltamethrin, amitraz, or cypermethrin in a comparative study, they showed sensitivity to cis-cypermethrin and cypermethrin only. Ovicidal activity of cis-cypermethrin, deltamethrin, and cypermethrin was assessed, and results showed that one day old eggs were more sensitive to these acaricides than 10-12 days old eggs. Additionally, different life stages (eggs, larvae, nymph, and adult) of *R. sanguineus* were also evaluated, with larval stages showing almost 100% mortality (Davey et al., 1989).

Synthetic pyrethroids are poorly soluble in water and highly lipid soluble. They have limited toxic effects against terrestrial animals because of the short life-span in the environment (Schleier III and Peterson, 2011). SPs have been shown to be carcinogenic when animals were exposed to them on a daily basis (US Environmental Protection Agency [USEPA], 2006).

## Synthetic Pyrethroids Mechanism of Action and Resistance

Voltage-gated sodium channels are integral transmembrane proteins, responsible for electrical propagation in most excitable cells. Sodium ions enter the cell by keeping the voltage-gated sodium channels open (activated) and cause membrane depolarization. The closure of voltage-gated sodium channels for just a few milliseconds after channel activation (fast inactivation) is responsible for the action potential’s falling phase (Dong, 2007). The mechanism of action of different SPs have been suggested to involve adhesion to the target site (voltage-gated sodium channels), causing these channels to remain open for a longer period, and resulting in a higher influx of sodium ions into the cell, with aperiodic neuronal discharge (Lees and Bowman, 2007). The voltage-gated sodium channel (containing four domains (I–IV) each having six segments) is a known SP target, where point mutations may be the cause of resistance in many tick species (He et al., 1999; Dong, 2007).

Type-I SPs cause the repetitive release of sodium ions in response to even a single stimulus, while SPs type-II cause membrane depolarization by interfering with membrane action potential. Type-II pyrethroids also delay the inactivation of voltage-gated sodium channels for longer periods compared with type-I SPs (Shafer et al., 2005).

Synthetic pyrethroids resistance is linked to mutations in voltage-gated sodium channel genes of *R. microplus*, with polymorphisms being identified in two of the four domains (domain-II and domain-III) as shown in **Table 2** (Morgan et al., 2009) (**Table 2**).

## Macrocyclic Lactones

Due to the unusual ability to eliminate both external and internal parasites, macrocyclic lactones are referred to as “endectocide” (Shoop et al., 1995). Avermectin, doramectin, selamectin, abamectin, ivermectin, eprinomectin, milbemycins, moxidectin, milbemycin oxime, and spinosyns are the MLs most commonly used in cattle (Rodriguez-Vivas et al., 2018). Different MLs have been used for the control of internal (gastrointestinal nematodes and microfilariae) and external (ticks, mites, and mange) parasites (Vercruysse and Rew, 2002). The use of a generic version of MLs, or excessive use of MLs in tropical and subtropical countries have influenced the selection of resistance in *R. microplus* (Perez-Cogollo et al., 2010a).

Environmental, biological, and management factors influence the selection of resistance against these acaricides in *R. microplus* populations (Perez-Cogollo et al., 2010b). The rise in potential resistance against ivermectin in *R. microplus* was observed after 10 generations in four different settings: larvae treated with ivermectin, larvae obtained from ivermectin-treated engorged female ticks, larvae obtained from ivermectin-treated engorged female ticks on an ivermectin-treated host, and larvae obtained from ivermectin-treated engorged female ticks that had high oviposition potential (Klafke et al., 2010). In *R. microplus* ticks collected from red deers, resistance against ivermectin was recorded for the first time in Mexico (Rodríguez-Vivas et al., 2014). In *R. annulatus*, resistance against ivermectin was studied for the first time in Egypt (El-Ashram et al., 2019). *Rhipicephalus microplus* ticks resistant against ivermectin have been reported from India (Singh et al., 2015b), while ivermectin-resistant *Hy. anatolicum* ticks were documented from Pakistan (Sajid et al., 2009; Kamran et al., 2021). Different MLs have different lipophilic properties, and human handlers should be careful to avoid contact with these drugs. Local swelling or pruritis at the injection site in animals occurs in response to subcutaneous administration of MLs. Additionally, the MLs treated animals suffer weight loss, increased body temperature and muscle tremors. Macrocyclic lactones, specifically ivermectin, become harmful to aquatic life when applied in water or when ivermectin-treated animals enter lakes, ponds, or streams shortly after application (Macrocyclic Lactones Veterinary-Systemic, 2006).

## Macrocyclic Lactones Mechanism of Action and Resistance

Protostome invertebrates, including ticks, express Glu-Cl channels in neurons and muscle, the target sites of MLs (Rodríguez-Vivas et al., 2010) (**Figure 3**). The mechanism of action of ivermectin was characterized by its high binding affinity to Glu-Cl receptors. These proteins control chloride ions movement across the channels located in the muscle and nervous cells (Cully et al., 1994), and thus their impairment can cause muscular paralysis and death of ticks by inhibition of neuronal impulses (Martin et al., 2002). When chloride gated channels remain open for a long period, conductivity of the membrane becomes irreversibly higher, which causes hyperpolarization as well as paralysis of tick muscles (Rodríguez-Vivas et al., 2010). Several species express efflux transporters called ABC-transporters (a super-family of integral membrane proteins). Extracellular and intracellular membranes can pump out the xenobiotic (drug, chemical, toxins) and endogenous metabolites through these protein channels, which reduces the concentrations of harmful chemicals in the cell. In *R. microplus*, these efflux pumps work as a defense mechanism against ivermectin (Pohl et al., 2011; El-Ashram et al., 2019), while esterases can also have a role in the detoxification of ivermectin (El-Ashram et al., 2019).

Macrocyclic lactones have been shown to be more effective against single host ticks than multi-host ticks (McKellar and Benchaoui, 1996 **).** Different studies (Martins and Furlong, 2001; Klafke et al., 2006; Castro-Janer et al., 2011) reported that *R. microplus* ticks are resistant to MLs, and mutations were observed in the Glu-Cl gene of *R. microplus* ticks (**Table 2**) (Aguilar-Tipacamú et al., 2016) (**Table 2**).

## Fipronil

Fipronil is a member of the phenylpyrazolic family of insecticides and has been used to control *R. microplus* infestations in cattle (Cole et al., 1993). While the use of fipronil was introduced in the late 1990s, resistance was only recently detected after the development of stable diagnostic tests (Cuore et al., 2007; Castro-Janer et al., 2009). It was persistently used in many countries and has been shown to be highly effective against ticks (Castro-Janer et al., 2009).

Fipronil was not widely utilized at first because of its higher costs relative to other chemicals. Its usage and demand increased in the early 2000s due to a decrease in costs and an increase in *R. microplus* tick populations resistant to other acaricides (Reck et al., 2014). *Rhipicephalus microplus* ticks collected in Mexico have developed resistance against fipronil, however, the extensive use of permethrin in the 1980s may have contributed to the selection of this resistance (Miller et al., 2013). *Rhipicephalus microplus* ticks resistant against fipronil have been reported from different regions such as Uruguay (Castro-Janer et al., 2009; Castro-Janer et al., 2010; Cuore and Solari, 2014), Switzerland (Lovis et al., 2011), and Brazil (Klafke et al., 2017; Becker et al., 2019). Other species of the genus *Rhipicephalus* (*R. sanguineus*) were also found to be resistant to fipronil (Samed et al., 2021). Fipronil-resistant *Hy. anatolicum* ticks were reported from Pakistan (Kamran et al., 2021). Resistance against fipronil may have developed at lower rates in *R. microplus* compared with *Hy. anatolicum* (Gupta et al., 2021). Fipronil is effective for tick control, but it is also toxic for honeybees, birds, fish and some other beneficial invertebrates. During fipronil applications, animals including humans (workers) can be exposed through their skin, eyes, or by breathing, resulting in skin irritation, sweating, nausea, vomiting, headache, stomach pain, dizziness, weakness, and seizures (Jackson et al., 2009).

## Fipronil Mechanism of Action and Resistance

Similar to OCs (e.g. lindane, dieldrin), fipronil acts by blocking GABA-receptors (Castro-Janer et al., 2009), and inhibiting the passage of chloride ions through GABA-gated chloride channels (**Figure 3**). It may cause mortality when used in high concentrations, while smaller doses may disrupt nervous functions (Cole et al., 1993). Fipronil is classified as a selective GABA antagonist because it binds effectively to the GABA-receptor chloride channels of ticks (Food and Agriculture Organization, 2009).

Mutations were also identified within the second transmembrane (TM-2) and third transmembrane (TM-3) domain of the GABA-gated chloride channel gene in fipronil resistant *R. microplus* ticks (Janer et al., 2019; Janer et al., 2021) (**Table 2**).

## Fluazuron

The fluazuron is a benzoylphenyl urea compound that inhibits chitin formation in *R. microplus*. Other benzoylphenyl urea compounds such as diflubenzuron, lufenuron, and flufenoxuron are effective against a wide range of insects except the fluazuron, which is effective against tick species (Junquera et al., 2019). Because of its narrow spectrum of activity and lack of lethal impact on established tick infestations, the fluazuron utilization was low to moderate after its introduction. However, its utilization has steadily increased due to the increasing resistance of *R. microplus* and *R. decoloratus* to numerous acaricides such as synthetic pyrethroids and amitraz (Food and Agriculture Organization, 2004), ivermectin (Martins and Furlong, 2001), and fipronil (Miller et al., 2013).

Fluazuron has shown higher efficacy against ticks than against other arthropods (Junquera et al., 2019). In Brazil, the field trials of fluazuron resulted in 99% effectiveness after treatment for 21 days (Martins et al., 1995b). Only larval stages of *R. microplus* ticks could be observed after the host was treated with fluazuron, and no ticks were found that had reached further developmental stages. Reduction in egg viability also occurred due to fluazuron application. Fluazuron has the ability to accumulate in fat, and may be released from the body through milk, thus protecting calves from ticks without direct application (Bull et al., 1996). It has been suggested that fluazuron affected solely the hatchability of larvae from treated female ticks, and that a synergistic effect of fluazuron with MLs negatively impacted reproductive parameters in *R. microplus* (Cruz et al., 2014). Fluazuron affected nymphs and larvae, when they were attempting molting, and did not harm any adult ticks (Junquera et al., 2019).

Fluazuron showed the highest efficacy (100%) than any other used acaricides against *R. microplus* ticks, and no resistance was reported against fluazuron, although it was suggested that its inappropriate use may lead to resistance development (Raynal et al., 2013). In a later study, resistance against fluazuron was recorded in *R. microplus* ticks (Reck et al., 2014).

## Fluazuron Mechanism of Action and Resistance

Fluazuron inhibits tick growth (Bull et al., 1996) by interfering with the formation of chitin through inhibition of enzymes involved in the molting process, and results in the hardening of the tick exoskeleton. Fluazuron treatment causes damage to several chitinous structures (smaller hypostome and chelicerae, scutum, sensilla, pores, anal plaque) in *R. sanguineus* nymphs, structures that play essential roles in tick survival (Calligaris et al., 2013).

Although the use of fluazuron was necessary for tick control and for cattle management, the careless use of this drug may weaken the immune system of the host, which in turn carries huge risks for diseases (Ribeiro et al., 2019). Although SNPs associated with fluazuron have not been reported, further research is essential to characterize its target site.

## Cross and Multi-Resistance against Different Acaricides

Cross-resistance between dieldrin (an OC) and fipronil has been reported, which affects the GABA-gated chloride channels of many insects (Le Corronc et al., 2002). The selection of cross-resistance against OCs led to a reduction in the use of these acaricides against different tick populations including *R. microplus, R. decoloratus*, and *R. appendiculatus* (Stone and Meyers, 1957; Baker and Shaw, 1965; George et al., 2004). It has been reported that there was a significant pattern of cross-resistance in many *R. microplus* populations against OCs (DDT) and SPs (cypermethrin) (Food and Agriculture Organization, 2004). Cross-resistance between two OPs (diazinon and coumaphos) and carbamate acaricide (carbaryl) has also been observed in *R. microplus* ticks (Li et al., 2005b). Cross-resistance between fipronil and lindane was examined in *R. microplus* ticks in Brazil and Uruguay, because both acaricides have the same target (Castro-Janer et al., 2010). *Rhipicephalus microplus* populations from Mexico were studied with multiple resistance to OCs, OPs and other acaricides (Foil et al., 2004; Alonso-Díaz et al., 2006).

## Mitigation of Acaricide Resistance and Alternative Control Methods

Strategies must be developed to minimize the selection of acaricide resistance in ticks. Different acaricides have different modes of action, thus alternate use may help in the reduction of resistance selection. Rotation of acaricides such as pyrethroids with coumaphos (OPs) has been used, and rotation of amitraz with other groups of acaricides may delay a further spread of amitraz resistance (Thullner et al., 2007). The alternate use of acaricides, especially amitraz with spinosad, may help in the minimization of resistance selection (Jonsson et al., 2010b). It has been recommended to perform further trials of experiments to finalize the conclusions about beneficial aspects of acaricide rotation in the field (Adakal et al., 2013).

Synergistic use of acaricide combinations may reduce the chances of resistance selection (Thullner et al., 2007), however, due to the lack of appropriate field work, this assumption remains to be confirmed. Nevertheless, the combination of fipronil with permethrin was recommended after showing effectiveness against *Dermacentor reticulatus* (Dumont et al., 2015b), *Ixodes ricinus* and *R. sanguineus* ticks (Dumont et al., 2015a). The combination of OPs (chlorfenvinphos and ethion) with SPs (deltamethrin and cypermethrin) has been successfully applied for the control of *R. microplus* (George et al., 2004). The combination of OPs (e.g. dichlorvos) with tetrachlorvinphos was successfully used against OP-resistant *R. microplus* (Davey et al., 2013). The combination of different acaricides such as flumethrin with cyfluthrin, chlorpyriphos with permethrin, and cypermethrin with cymiazole were available in the market, and were used for tick control in Mexico (Rodriguez-Vivas et al., 2006a). Satisfactory results (high larvae mortality rate) were observed from the combined effects of permethrin and amitraz, while there was zero larvae mortality when permethrin was used alone (Fernández-Salas et al., 2012). The application of combined acaricides may help in delaying the resistance selection, because individual ticks are very unlikely to have resistant alleles of two or more genes for different acaricides with different modes of action (Lovis et al., 2013). Deltamethrin, thymol, and essential oil derived from *Eucalyptus* have shown synergistic acaricidal efficacy against *R. microplus* (Arafa et al., 2021).

Besides resistance development, excessive use of acaricides has negative impacts on the environment, animal health, and animal-derived products. For these reasons, environmentally friendly alternative control strategies are needed, which would also be important for the mitigation of acaricide resistance. Numerous plant-derived products have been used to aid tick control. *Rhipicephalus microplus* ticks were killed at a nearly 100% rate by applying the essential oils of *Eucalyptus citriodora* and *Eu. staigeriana* at 10% concentration, while *Eu. globulus* showed 100% efficacy at 20% concentration (Chagas et al., 2002). In another study, *Eucalyptus* spp. leaf extracts showed 96% effectiveness against female *R. microplus* ticks (Costa et al., 2008). Larval mortality of 100% and 98%, and reproductive efficiency inhibition of 100% and 91.5%, were observed in *R. microplus* treated with *Melia azedarach* chloroformic and hexanic extracts, respectively (Borges et al., 2003). The acaricidal activity of environment friendly essential oils from different plant species has been tested against different ticks. Essential oils obtained from *Origanum bilgeri* and *Cunila* species have shown acaricidal activity against *Rhipicephalus* ticks (Apel et al., 2009; Koc et al., 2013). Thymol, a major component of essential oils, has shown 100% acaricidal efficacy against *R. microplus* and *R. sanguineus* ticks (Daemon et al., 2009). Essential oils extracted from *Allium sativum* were 90-100% effective in killing *R. microplus* larvae (Martinez-Velázquez et al., 2011). Essential oils extracted from *Tagetes minuta* have shown 95% efficacy against larvae of *Amblyomma cajennense* and *Argas miniatus* (Garcia et al., 2012). The application of essential oils extracted from *Azadirachta indica* seeds affected egg hatching and caused 100% larval mortality in *Hy. anatolicum* ticks (Abdel-Shafy and Zayed, 2002). Oils extracted from leaves of *Hesperozygis ringens* caused 95% inhibition of larval hatchability and 100% larval mortality in *R. microplus* (Ribeiro et al., 2010). Two monoterpenes (carvacrol and thymol) were used alone or in combination with cypermethrin to control *R. microplus.* It was observed that thymol enhances cypermethrin toxicity (Tavares et al., 2022b), and that both monoterpenes increased the activity of antioxidant and detoxifying enzymes (glutathione-S-transferase, catalase, superoxide dismutase, and glutathione peroxidase) in the *R. microplus* ticks (Tavares et al., 2022a). The combination of essential oils from *Cinnamomum zeylanicum*, *Cuminum cyminum*, and *Pimenta dioica* in emulsion preparations showed a synergic effect against *R. microplus* (Lazcano Díaz et al., 2019). Furthermore, the combination of essential oils extracted from *Laurus nobilis* and *Copaifera officinalis* showed higher efficacy than the isolated oils against *R. microplus*, indicating that the synergic action could increase the effect of environment-friendly compounds (Vinturelle et al., 2021). Some of the mechanisms of acaricidal activity by plant products include inhibition of egg development, suppression of chitin formation, inhibition of growth regulator hormones, and disruption of the mating communications. These products have different modes of action, thereby slowing down the selection of resistant ticks (De Souza Chagas et al., 2012; Pavela et al., 2016). Factors such as identification of suitable phyto-metabolites, extraction time of phytochemicals, the concentration of extracts, odors of plant-derived extracts, extracted plant age, specie of ticks, and exposure time of ticks may have undermined the research on phytotherapeutic alternatives. During many years, various studies were focused on the identification of natural resources, but recent research has focused on chemical standardization, quality of phytochemicals for tick control, and other issues like long-term stability, storage, and transportation of different plant extracts, which are essential for the commercial availability of these products (Benelli and Pavela, 2018; Selles et al., 2021).

There are reports of 58 species of fungi found infecting 73 different acari, and several fungi species have shown potential anti-tick efficacy. *Beauveria bassiana, Metarhizium anisopliae, Isaria farinosa*, *Isaria famosorosea*, and *Lecanicillium lecanii* have been shown to infect various ixodid ticks (Chandler et al., 2000; Jackson and Jaronski, 2009). Environmental factors that affect the persistence and virulence of fungi against ticks include high or low environmental temperature, low humidity, UV radiation, precipitation, soil type, composition and pH (in case of direct application on soil) (Jackson et al., 2009; Jaronski, 2010; Wu et al., 2020). The stability of some fungal products is very limited, which is why its large-scale production can be expensive. Most manufacturers would recommend exactly the same application method for fungal products as used for other chemical pesticides (Shelton and Roush, 2000).

To minimize the need for acaricides and delay resistance selection, the strategic use of anti-tick vaccines has been strongly and preferably recommended as suitable alternatives for tick control (Guerrero et al., 2012b). The first commercial anti-tick vaccine was developed by expressing gut glycoproteins (Bm86) of diversely distributed *R. microplus* (Willadsen and Kemp, 1988). The effectiveness of this anti-tick vaccine varies in different regions against different ticks, therefore the identification of novel antigens as vaccine candidates are underway (Freeman et al., 2010). Since Bm86, numerous novel antigens from several tick species have been tested for potential anti-tick vaccine development, however, none has been approved for the market so far (Parizi et al., 2012; de la Fuente et al., 2017; Rego et al., 2019; Ali et al., 2022).

The development of alternative approaches to mitigate tick resistance against available acaricides, such as the implementation of non-chemical control methods, application of biosafety protocols for the prevention of resistant ticks, development of novel environment-friendly acaricides, suitable diagnostic methods, and anti-tick vaccines, are in continuous and pressing demand.

## Conclusion

All of the available acaricides have faced failure due to the selection of tick resistance. The repeated use of a single acaricide is often responsible for the selection of fast-developing acaricide resistance. Regular surveillance of ticks for resistance against different acaricides may play a vital role in the minimization of resistance selection. Livestock owners and workers must be trained on tick control and resistance mitigation measures. Environmentally friendly, stable, cheaper, easily accessible, and novel acaricides, as well as anti-tick vaccines should be developed to overcome tick resistance against acaricides.

## Author Contributions

MKO, NI, AAlo, AZK and AA searched and collected the literature. ISV, TT, AA supervised the overall investigations. All authors equally contributed to the formulation of the whole manuscript.

## Funding

We acknowledge the financial support provided by Pakistan Science Foundation and Higher Education Commission Islamabad Pakistan, researchers supporting project number (RSP2022R494), and the CNPq and CAPES – Brazil for providing research facilities. This work was supported by JSPS KAKENHI Grant Number JP22H02522.

## Conflict of Interest

The authors declare that the research was conducted in the absence of any commercial or financial relationships that could be construed as a potential conflict of interest.

## Publisher’s Note

All claims expressed in this article are solely those of the authors and do not necessarily represent those of their affiliated organizations, or those of the publisher, the editors and the reviewers. Any product that may be evaluated in this article, or claim that may be made by its manufacturer, is not guaranteed or endorsed by the publisher.
